# Intrathoracic Extrapulmonary Pleomorphic Liposarcoma: A Rare Presentation

**DOI:** 10.7759/cureus.86018

**Published:** 2025-06-14

**Authors:** Peter N Rodenko, Josh S Elefteratos, Colton A Herrell, Emily L Rodenko, Timothy Townsend

**Affiliations:** 1 Medicine, St. George's University School of Medicine, St. George, GRD; 2 Internal Medicine, St. George's University School of Medicine, St. George, GRD; 3 Biology, Trinity University, San Antonio, USA; 4 Radiology, Medical Center Health System, Odessa, USA

**Keywords:** intrathoracic extrapulmonary liposarcoma, intrathoracic extrapulmonary pleomorphic liposarcoma, intrathoracic pleomorphic liposarcoma, liposarcoma, pleomorphic intrathoracic extrapulmonary liposarcoma, pleomorphic liposarcoma, pleural liposarcoma, rare presentation pleomorphic liposarcoma, young adult liposarcoma, young adult pleomorphic liposarcoma

## Abstract

Liposarcoma (LPS) is a malignant mesenchymal neoplasm originating from adipocytes. LPS comprises several histological variants, among which pleomorphic liposarcoma (PL) constitutes a rare and highly aggressive subtype. PL is most commonly found in the extremities and presents primarily in individuals aged 50 and above. Primary thoracic presentations of PL are exceedingly rare, particularly in young adults, and are often associated with metastases to the lungs. This case reports a 28-year-old white male with no significant past medical history presenting with shortness of breath, left posterior thoracic pain, and a dry cough. Imaging revealed a large intrathoracic, extrapulmonary mass arising in the left pleura with compression of the heart and ipsilateral lung, resulting in atelectasis, but without evidence of chest wall or pulmonary invasion. Hemothorax, empyema, or pleural effusion were among the differentials initially suspected due to the location and density of the abnormality; however, drainage only yielded scant fluid.

Thoracotomy for pleural decortication later revealed a solid mass with local pleural adherence. Histopathology of tissue biopsies confirmed a high-grade pleomorphic liposarcoma with extensive necrosis and pleomorphic lipoblasts. The patient was started on the AIM (adriamycin/doxorubicin, ifosfamide, mesna) regimen, and care was transferred for further oncologic management. Many features of this rare liposarcoma subtype were highly unusual, including the age of presentation, tumor location, lack of extensive lung invasion or metastasis, and absence of known genetic or environmental risk factors. Given the poor prognosis associated with PL, as well as its highly aggressive nature, treatment generally requires multimodal management that often challenges existing treatment frameworks. This is in part due to the relative deficit of literature regarding PL compared to other LPS subtypes. This case not only demonstrates an unlikely sarcoma presentation but also emphasizes the therapeutic limitations in managing pleomorphic liposarcoma and the necessity for further research into subtype-specific treatment strategies.

## Introduction

Liposarcoma (LPS) is a malignant mesenchymal tumor of lipoblasts or adipocytes that is rare among other neoplasms. It is the most common malignant tumor of soft tissues, representing up to 20% of soft tissue sarcomas [1]. Depending on the subtype, these malignancies can arise from deep soft tissues in many anatomical locations, including the extremities, retroperitoneum, mediastinum, paratesticular regions, head, neck, and esophagus [2]. It is important to note that benign lipomas have an extremely low probability of progressing to liposarcomas [3]. 

There are four major types of liposarcomas: atypical lipomatous tumor (ALT)/well-differentiated liposarcoma (WDL), dedifferentiated liposarcoma (DDL), myxoid liposarcoma (ML), and pleomorphic liposarcoma (PL) [2]. ALT/WDL and DDL are the most common subtypes and involve a well-documented 12q14-15 gene amplification [2]. ALT is differentiated from WDL according to how resectable the tumor is based on location, with ALT arising from more surgically accessible anatomical locations, such as the extremities, from which curative resection can be performed [4]. LPS would be classified as WDL when presenting in a surgically inaccessible area where tumor invasion would be more likely to occur such as the retroperitoneum, paratesticular regions, or mediastinum [4]. ML, the third most common subtype, typically arises in the extremities and has a well-understood mutation in chromosome 12 commonly featuring a FUS-CHOP fusion [5]. DDL, which is genetically associated with the same 12q14-15 amplification as ALT/WDL subtypes, most frequently arises from the retroperitoneum and can occur de-novo or after recurrence of previous ALT/WDL [6]. PL is a rare and karyotypically complex liposarcoma subtype that has variable clinical outcomes.

Pleomorphic liposarcoma accounts for under 5% of LPS cases and is the least understood major subtype in current literature, mostly being represented by studies starting in the twenty-first century [7-9]. PL typically occurs in the fifth to seventh decades of life and most commonly originates in the extremities, with a predominance in the lower extremity soft tissues [8]. PL has been most commonly linked to TP53, RB1, and NF1 mutations, but this subtype is also associated with variable chromosomal aberrations and is more genetically and morphologically complex than other LPS subtypes, which have relatively well-understood mutations [2]. Due to these complexities combined with the paucity of literature on targeted therapies, PL has the worst prognosis of LPS subtypes, with a five-year survival rate of 57% [7]. While PL typically presents in the extremities of elderly patients with secondary lung metastasis [2], this case describes an unusual presentation of a primary intrathoracic, extrapulmonary pleomorphic liposarcoma in a young adult.

## Case presentation

This case involves a 28-year-old white male with a past medical history of asthma and attention deficit hyperactivity disorder presenting to the emergency department with worsening shortness of breath. The shortness of breath had been present for three months but acutely worsened two days prior to presentation. There was an associated sharp back pain that started acutely two days prior and was located in the left posterior thoracic region without radiation. There was also an associated non-productive cough that started three days prior to admission. The patient took no medication regularly and had no significant smoking, alcohol, or drug use history. There were no occupational exposure risks, and there was no known family history of major disease or cancer. The patient’s asthma had been well-controlled and had not caused any symptoms since childhood. The patient denied extremity swelling, claudication, palpitations, nausea, vomiting, abdominal pain, fatigue, or syncopal episodes. The patient had never experienced a similar episode.

On physical exam, the patient appeared in mild distress but was alert. The patient was tachycardic at 130 beats per minute, had an oxygen saturation of 87%, and otherwise had stable vitals. Respirations were mildly labored, and breath sounds were clear bilaterally on auscultation with mild tachypnea. The electrocardiogram demonstrated sinus tachycardia. Initial labs are exhibited in Table 1, indicating normocytic anemia, a mildly elevated prothrombin time, and leukocytosis. Additionally, Chem 6 panel results for CO2 (carbon dioxide) content, chloride, creatinine, potassium, sodium, and urea nitrogen were within normal ranges.

**Table 1:** Relevant lab values

Relevant Labs	Lab Values	Normal Reference Values
Hemoglobin (g/dL)	11.8	13.2 – 16.6 (males)
Total Iron Binding Capacity (mcg/dL)	240	250 – 450
Prothrombin Time (seconds)	16	11 – 15
Leukocytes (cells/microliter)	14,800	4,500 – 11,000
Troponins (ng/mL)	0.1	0 – 0.4

**Figure 1:**
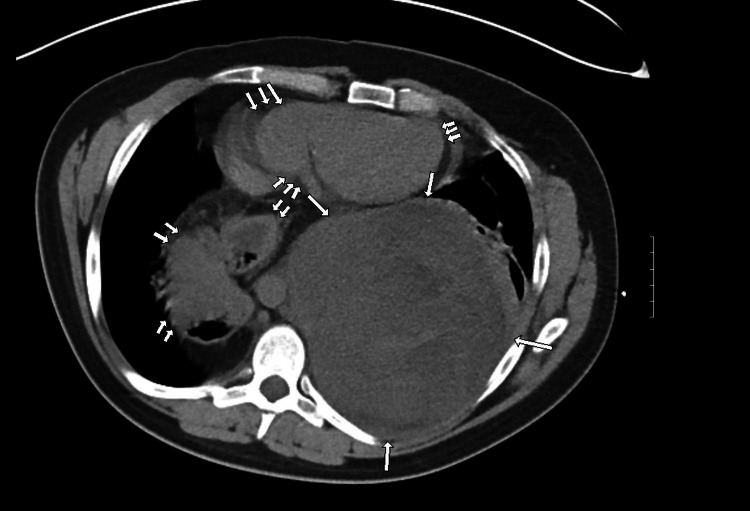
Non-enhanced chest CT axial image of the lower chest with a soft tissue window (with arrows): a large intrathoracic extrapulmonary mass compressing the heart is seen (single arrow). An incidental hiatal hernia is also visualized in the right lung space (double arrows). The heart is compressed anteriorly by the mass against the chest wall (triple arrows). The abnormality does not contain fat density when compared to abdominal fatty regions. Hounsfield units (HU) range from 5 to 30 HU with an average of 25 HU across the abnormality.

**Figure 2:**
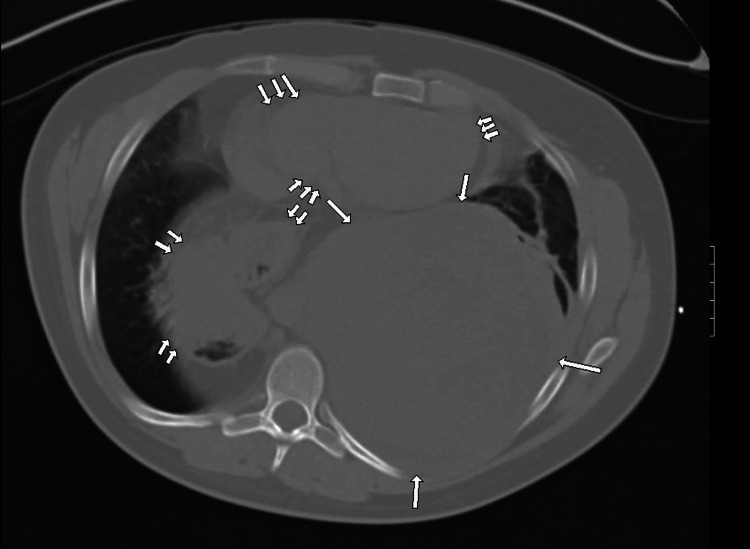
Non-enhanced chest CT axial image of the lower chest with lung window (with arrows) demonstrating the same findings noted in Figure 1 with a more detailed visualization of the air spaces

A chest CT without contrast (represented by Figure 1 and Figure 2) was ordered, showing a left posterior, intrathoracic, and extrapulmonary abnormality. The abnormality was large in size, heterogeneous in composition, and of intermediate density. The lesion displaced and mildly compressed the heart anteriorly. It also displaced the left lung anteriorly with atelectasis accentuated by a large hiatal hernia containing stomach and colon segments. This lesion was projected to be in the pleural space with no obvious chest wall involvement.

The non-fatty and heterogeneous characteristics of the mass with an average of 25 HU on imaging suggested hemothorax, empyema, and soft tissue mass as differentials at the time. Due to the lack of an air-fluid level and the blood density of the abnormality, hemothorax was considered to be the most likely cause, indicating the need for percutaneous drainage. Infectious Disease recommended a seven-day course of Zosyn, protecting for possible empyema or parapneumonic effusion. Percutaneous thoracostomy drainage was attempted and yielded a scant amount of bloody effluent, likely due to clot formation and hardening of the suspected hemothorax. Subsequently, thoracotomy for pleural evacuation and decortication was performed, which unexpectedly revealed a solid extrapulmonary mass in the posterior chest with total compression of the left lower lung lobe and adherence to the pleura of the left upper and lower lung lobes, possibly indicating a small degree of local invasion. Dark blood and necrotic material oozed voluminously from the solid mass, and there was an estimated 3.5 L of blood loss intraoperatively. The mass was partially resected during the thoracotomy procedure, and biopsies of the sample were taken for histologic evaluation.

The pathology report showed high-grade liposarcoma with extensive necrosis, occasional giant cells, and pleomorphic lipoblasts with numerous mitoses. The samples were strongly positive for vimentin and negative for pankeratin. The results were diagnostic of a pleomorphic liposarcoma with possible differentials of other dedifferentiated sarcomas. The patient was started on five cycles of adriamycin, ifosfamide, and mesna (AIM) every 21 days, as well as pegfilgrastim for immunoprotection. The patient transitioned care to another hospital’s oncology service and is currently finishing the AIM regimen. After completion of chemotherapy, the patient will undergo further imaging to evaluate tumor responsiveness to treatment.

## Discussion

This case presents a young adult patient with a primary pleomorphic liposarcoma of thoracic origin, possibly arising from the pleura but not involving the chest wall or extensive metastasis to the lungs. The rarity of this case cannot be overstated, as <1% of primary liposarcomas are present within the thoracic cavity, and under 1% of mediastinal tumors are found to be liposarcomas [4,10]. Of the minuscule fraction of liposarcomas that originate in the thoracic cavity, most have extensive metastasis [2,10]. This contrasts with this patient’s tumor presentation, which showed minimal metastatic pulmonary invasion on further investigation, suggesting that his pulmonary symptoms were due to the mass effects of the tumor. The patient likely experienced the presenting symptoms due to compression from the tumor on the heart anteriorly in conjunction with local invasion and irritation from the mass on the somatic pleural lining. These clinical manifestations align with the slow-growing nature and typical symptomatic course of liposarcomas [10], progressing from an asymptomatic presentation to symptoms that were consistent with compressive atelectasis and mediastinal impingement.

The anomalous nature of this patient’s liposarcoma is highlighted not only by the location in which it presented but also by the subtype and age at which the patient was diagnosed. Liposarcomas most commonly present in elderly patients, yet this patient is a young adult [8]. His other social demographics were consistent with the typical patterns seen in LPS prevalence, as white males are more frequently predisposed to this neoplasm [11]. Moreover, of the pediatric and young adult liposarcomas recorded, the vast majority are of the ML subtype, while this patient presented with the less common PL subtype [12]. Of note, the pathology report mentioned the potential presence of other DDL variants due to the recent expansion of the DDL morphological spectrum to include rare variants. This distinction is important because testing additional biopsy samples would rule out dedifferentiated variants, further supporting a PL diagnosis [7].

Few cases of liposarcoma have presented in the thoracic cavity with minimal metastatic pulmonary invasion and few associated risk factors as seen in this patient. This patient has no known family history of Li-Fraumeni syndrome, retinoblastoma, neurofibromatosis 1, and other genetic anomalies associated with soft tissue sarcomas, which are most commonly associated with LPS development among other types of neoplasms [2]. He does not smoke or have any known exposure to toxic chemicals, industrial solvents, or radiation. In the absence of significant risk factors or features commonly associated with liposarcomas, the pathophysiological mechanism of this patient’s pleomorphic tumor remains unclear. Further genetic testing for these associated mutations may help understand the underlying mechanism predisposing this patient to LPS. 

Pleomorphic liposarcomas only account for 5-10% of all lipomatous sarcomas, yet present as the most aggressive, metastatic, and deadly subtype [9]. PL are known to have rapid, distant metastasis to the lungs (>50%); however, this patient’s tumor developed in the thoracic cavity without significant permeation into the lungs [13]. Early surgical resection of the tumor, followed by chemoradiation, results in the highest likelihood of remission, but the local recurrence rate for this LPS subtype is about 45% [13]. Given the elevated risk of recurrence, the surgical intervention often involves radical resection of entire organs in the affected region (most commonly the kidneys or sections of the colon) [14]. This results in a significantly increased risk for subsequent complications and often leads to substantial deterioration in patients’ quality of life. New methodologies of preventing the recurrence of PL while reducing the extent of resection would most likely benefit patients’ quality of life and reduce iatrogenic complications and mortalities. Current efforts for unresectable LPS are focused on preventing recurrence and metastasis with systemic chemotherapies, though the role of chemotherapy in these cases is often limited [14]. This patient was put on the AIM (adriamycin, ifosfamide, mesna) regimen, which has been shown to be effective in managing soft tissue sarcomas [15]. While effective, this regimen is a broad therapy used for advanced or unresectable soft tissue sarcomas, but data are insufficient on well-established and targeted therapies for PL due to its morphologically and genetically heterogeneous nature [16]. The mainstay of PL chemotherapy involves adriamycin/doxorubicin or ifosfamide-focused therapies, but there is evidence to suggest that pegylated liposomal doxorubicin and ifosfamide combination therapy results in a lower rate of treatment-related toxicity with similar efficacy compared to the standard regimen [17]. Evolving systemic therapies with eribulin and trabectedin have shown encouraging results in comparison to doxorubicin monotherapy or combination therapy [15].

## Conclusions

The case presents a unique instance of pleomorphic liposarcoma (PL) in a young adult arising intrathoracically within the pleura and with minimal involvement of the lungs. The age at presentation, combined with tumor location and morphologic characteristics, was highly uncommon. The absence of known genetic, environmental, or familial risk factors further underscores the atypical nature of this case, highlighting a critical gap in understanding the etiology and progression of PL in such cases. While regimens like AIM (adriamycin/doxorubicin, ifosfamide, and mesna) have proved to be broadly effective in treating soft tissue sarcomas, the paucity of targeted therapies for PL due to this sarcoma’s genetic complexity and aggressive nature limits its long-term treatment options. This case demonstrates an atypical presentation of an already enigmatic pathology, emphasizing the need for targeted therapeutic strategies to improve morbidity and quality of life in future PL cases.
